# Insulin Therapy and Body Weight, Body Composition and Muscular Strength in Patients with Type 2 Diabetes Mellitus

**DOI:** 10.1155/2010/340570

**Published:** 2009-10-21

**Authors:** H. Gin, V. Rigalleau, C. Perlemoine

**Affiliations:** ^1^UMR 5536 CNRS/Université de Bordeaux 2(Victor Segalen), 33000 Bordeaux, France; ^2^Service de Nutrition-Diabétologie, Hôpital Haut-Lévêque, Avenue de Magellan, 33600 Pessac, France

## Abstract

*Aims.* To determine the progression of body weight (BW) and body composition (BC) in patients with type 2 diabetes mellitus (T2D) on insulin therapy and the consequences on muscle strength (MS) as a reflect of free fat mass increases. *Research design and methods.* We analysed BC using air displacement plethysmography and MS by hand grip dynamometry in 40 T2D before and after three (M3) and six months (M6) of insulin therapy. *Results.* at baseline HbA1c was 9.76 ±1.6% and BW was stable with fat mass (FM) 28 ± 10.7 kg; and fat free mass (FFM) 52.4 ± 11 kg; at M6, HbA1c improved to 7.56 ± 0.8%; insulin doses tended to increase. BW gain at M6 was + 3.2 ± 4.2 kg and with an increase of only 25% by M3; it was composed of FM, whereas FFM was unchanged. MS did not increase on insulin therapy. *Conclusions.* In T2D, BW gain was composed exclusively of FM with no improvement in MS.

## 1. Introduction

Insulin is a major therapeutic tool for insulin-deficient patients with type 1 diabetes mellitus, and also for patients with type 2 diabetes mellitus (T2D), with both insulin resistance and a relative insulin deficiency [1]. Its effects on glucose metabolism are well known, but insulin also influences lipid and protein metabolism with anabolic and anticatabolic effects. These have been less studied despite body weight (BW) gain being an important consequence of insulin therapy, identified in both the DCCT [2] and UKPDS [3] studies. BW gain is probably a benefit in purely insulin-deficient patients in whom lack of insulin is responsible for lean tissue loss [4], but in T2D the worsening of existing obesity seems undesirable.

Analysis of body composition (BC) changes can help determine whether insulin-induced BW gain can be considered as beneficial or not, by distinguishing the accumulation of fat (FM) from that of fat-free mass (FFM). FFM is an important nutritional parameter, linked with immune competence, functional status, and survival [5]. Previous studies on the influence of insulin therapy on BC of patients with diabetes mellitus have been limited because the methods for analyzing BC (tritiated water, underwater weighing or four-compartment model) are difficult to use in clinical practice. Air displacement plethysmography is a new, safe, quick, and valid technique, providing accurate measurements of body density [6] and discriminating moderate changes in FM and FFM [7]. Although some uncertainties due to the hydration of FFM may influence any two-compartment model-based analysis [8], the simultaneous measurement of muscle strength (MS) by dynamometry can be used to confirm the results of FFM: a correlation between FFM and MS has been previously demonstrated [9–11] 

The aim of our study was to assess the effects of insulin therapy on body weight and composition, and on muscular strength, in T2D with failure to oral hypoglycaemic agents.

## 2. Subjects and Methods

### 2.1. Patients

40 type 2 diabetic patients (18 men and 22 women) who presented secondary failure to oral antidiabetic agents with HbA1c > 7% after a duration of 13.5 ± 8.7 years of diabetes mellitus were recruited during their stay in our department. The only inclusion criterion was the introduction of insulin. Exclusion criteria included a reduced autonomy, which could interfere with the plethysmographic measurement, and any condition leading to water and salt retention or any severe concurrent illness.

All T2D were treated with insulin injections and their oral antidiabetic agents. All patients monitored their blood glucose and were asked to adapt the insulin doses in order to obtain HbA1c close to recommended levels [12]. They were advised during their hospitalization about diet and physical activity.

All patients were fully informed of the purpose of the study and gave their informed consent to take part in the investigation. The university hospital ethics committee accepted the protocol.

### 2.2. Study Design

At baseline, all patients underwent a physical and laboratory examination to rule out any undercurrent disease. When insulin therapy was indicated, the patients were invited to participate in the study and all accepted. We carried out BC analysis and MS measurements at baseline, then 3 and 6 months later. The baseline analysis was conducted just before starting insulin.

### 2.3. Analytical Methods

HbA1c was measured on EDTA Vacutainer samples by affinity chromatography using a Hi-AUTOA1c analyzer (A Menarini Diagnostics, Antony Cedex, France) initially and 3 and 6 months later.

BW was measured on an electronic scale to the nearest 0.1 kg and height to the nearest 0.1 cm. Waist circumference (WC) at the umbilical midline was measured to the nearest mm with flexible tape. 

Air displacement plethysmographic measurements were carried out using the Bod-Pod body composition system (Life Measurement, Inc, Concord, Calif, USA) according to the manufacturer's recommendations [13]. The procedure involved the calibration of the system when empty and then when a 49.771-liter metal cylinder was placed inside. After being weighed on an electronic scale, the patients, in their undergarments and wearing a swimming cap, were seated inside the chamber and asked to remain with hands positioned on their thighs and breathing normally. Body volume was assessed by the difference between the volume of air inside the chamber when empty and then with the subject present. If two consecutive measurements of volumes differed from each other by more than 150 mL, a third measurement was conducted. Body density was calculated as Body Weight/Body Volume and fat mass was calculated from the Siri equation (%FM = (4.950/Body density)-4.500)×100 [14]. Predicted respiratory volumes were taken into account in the calculations.

Three consecutive measurements of handgrip strength with the dominant hand were performed with a calibrated dynamometer (Takei Scientific Instruments, Tokyo, Japan), which was reset to zero before each measurement. The measurements were conducted under standardized conditions: subject seated, the shoulder adducted and neutrally rotated, with the elbow at 90° flexion and the forearm and wrist in a neutral position. Patients were encouraged using a standard phraseology (squeeze the handle as hard as possible). Mean values were recorded.

### 2.4. Statistical Analysis

Data are shown as means with standard deviations. SPSS software 10.0.5 was used for the calculations (standard version, copyright Ó SPSS Inc. 1989–1999). A one-way analysis of variance for repeated measures over time (ANOVA) was used to compare the values at baseline (M0), 3 months (M3), and 6 months (M6). Associations were tested by linear regression. *P* < .05 was considered significant.

## 3. Results

### 3.1. Baseline

The T2D were 62 ± 12 years, their BW was stable over the previous three months: BW 85.4 ± 14.7 kg: BMI 30.9 ± 5.7 kg/m^2^, with high FM 28 ± 10.7 kg, and WC: 105.7 ± 12 cm,. FFM was 52.4 ± 11 kg; and MS was 29.1 ± 8.2 kg; Glycaemic control was poor: HbA1c 9.76 ± 1.6%.

### 3.2. Progression With Insulin Therapy (Table 1)

**Glycaemic Control and Insulin Doses**

HbA1C improved during the first three months, and then stabilized. M6 HbA1c: 7.56 ± 0.8%. The doses of insulin tended to increase from M0 to M6.

**Body Weight and Composition**

BW increased on insulin treatment to 88.5 ± 14 kg, and 75% of the increase occurred after 3 months. The composition of the BW gain: during the first three months FM and FFM were unchanged, but at six months, FM increased* (* *P* < .012(M6/M0))whereas FFM remained unchanged, BW gain was related to changes in FM (*r* = 0.83, *P* < .0005) and WC (*r* = 0.81, *P* < .0005), but not to those of FFM(*r* = 0.12, *P* = .63). BW gain was related to the initial HbA1c (*r* = 0.70, *P* < .001), to the decrease in HbA1c (*r* = −0.61, *P* = .007), and to the insulin doses (*r* = 0.81, *P* < .0005) in T2D.

### 3.3. Muscle Strength

MS was correlated with FFM (*r* = 0.72, *P* < .0005) and inversely correlated with FM (*r* = −0.52, *P* = .001). MS remained stable and did not increase during the six months.

## 4. Discussion

In this study, we studied the progression of BW, BC, and MS during six months after starting insulin therapy in 40 T2D. Insulin induced a significant decrease in HbA1C, that approached the recommended level in type 2 diabetes (7.5± 0.9%). The course and nature of the BW gain was +3.1 ± 2.2 kg at M6 and 75% of the increase occurred after M3. All the weight gain was due to FM (+3.1 ± 2.7 kg), whereas FFM was unchanged (−0.1 ± 1.6 kg) and T2D did not gain MS on insulin therapy. The BW gain at M6 was correlated with initial HbA1c and insulin doses and with the decrease in HbA1C. 

One limitation of our study stems from the use of a two-compartment model for the analysis of BC by air displacement plethysmography (ADP), as hydration of FFM cannot be assumed to be constant during insulin therapy. Overall our results are in agreement with previous studies. In T2D, previous studies reported that BW gain (+1.6 ± 4.9 to +5.2 ± 2.7 kg at six months) was mainly composed of fat [15–20]. We confirm the predominance of FM gain in insulin-treated T2D as it represented the entire BW gain; we demonstrated for the first time the absence improvement in MS after insulin therapy in type2 diabetic patients that confirm the absence of improvement in FFM during weight gain in insulin treated T2D. Higher MS in type 1 diabetic patients versus T2D has been reported by Cetinus et al., as compared to age-matched control subjects [21]. The correlation between the decrease in HbA1c and BW gain, and with insulin doses have been reported by other authors [18, 20]. 

Our study did not aim to explain the progression of BC during insulin treatment, but several studies investigating the role of insulin on protein metabolism in diabetes mellitus have provided useful data. In type 1 diabetes, protein turnover studies using isotopically labeled amino acid tracers have demonstrated that protein breakdown and amino acid oxidation are increased in the insulin-deprived state, and are normalized by insulin, which has an anticatabolic effect [4]. The effect of insulin on protein synthesis is more difficult to explore and a matter of debate: in type 2 diabetic patients, the effect of acute administration of insulin on suppression of muscle protein breakdown was noted [22], but a resistance to insulin action develops on chronic administration [23]; a reduced nutritive blood flow may be involved in the resistance of muscular protein synthesis to the action of insulin in type 2 diabetic patients; indeed, the response of muscle protein synthesis is impaired in the elderly [24], due to the inability of insulin to increase vasodilatation [22]; adiposity is also associated with a reduced whole-body protein anabolic action of insulin in women [25] and the elderly [26]. So in type 2 diabetic patients, insulin therapy can be without any effect on FFM and consequently on MS. 

Insulin therapy is clearly beneficial for glycemic control in T2D but we do not feel that the gain in FM offsets this benefit. However, the excess fat is deposited essentially in the intra-abdominal area [20]. Although we did not use DEXA to determine the distribution of FM gain, we observed an increase in WC, which is a good indicator of visceral FM [27]. The long-term consequences of these nutritional changes need to be determined.

In summary, BW gain of TD2 on insulin therapy was 3.1 ± 2.2 kg, it was predominantly fat with no improvement in muscular strength or FFM.

## Figures and Tables

**Table 1:** Evolution from M0 to M6 of body parameters and Hb1Ac levels during insulin therapy in patients with type 2 diabetes mellitus. Results are mean (SD), *P* indicates the significance of the difference versus the baseline value (NS: nonsignificant).

	3 months (M3)	6 months (M6)
Weight change (kg)	+ 0.7 (2.3)	+ 3.1 (2.2) *P* < .008
Fat mass change (kg)	− 0 .2 (3.1)	+ 3.1 (2.7) *P* < .012
Waist circumference change (cm)	−0.3 (3.8)	+ 1.7 (4.5) *P* < .004
Fat free mass change (kg)	+ 0.9 (2.9) *P* = .15 (NS)	− 0.1 (2.6) *P* = .82 (NS)
Muscle strength change (kg)	+ 0.4 (2.9) *P* = 0.57	+ 0.4 (4.2) *P* = .87
HbA1c change (%)	− 2.4 (1.7) *P* < 0.0005	− 2.2 (1.7) *P* < .0005
Insulin doses (U/kg)	0.41 (0.32)	0.44 (0.27)

## Abbreviations

ADP: Air displacement Plethysmography
BC: Body composition
BW: Body Weight
FFM: Fat-free mass
FM: Fat mass
MS: Muscular strength
T2D: patients with type 2 diabetes mellitus
WC: Waist Circumference.
